# Cardiometabolic and RAAS-Targeted Therapy in Thoracic Aortic Aneurysm: Propensity-Matched Associations with Survival and Major Cardiovascular Events

**DOI:** 10.3390/medsci14020329

**Published:** 2026-06-18

**Authors:** Hussein Abdul Nabi, Luke Dreher, Soad Al Osta, Fadi E. Shamoun

**Affiliations:** Department of Cardiovascular Medicine, Mayo Clinic Arizona, 5777 East Mayo Blvd., Phoenix, AZ 85054, USA

**Keywords:** thoracic aortic aneurysm, major adverse cardiovascular outcomes, survival outcomes, GLP-1 receptor agonists, SGLT-2 inhibitors, ACE inhibitors, angiotensin receptor blockers (ARBs)

## Abstract

Background: Thoracic aortic aneurysm (TAA) remains a high-risk vascular condition despite major advances in imaging surveillance, operative repair, and endovascular therapy. Medical management still relies largely on blood pressure control and global cardiovascular risk reduction. Renin–angiotensin–aldosterone system (RAAS) inhibitors are frequently used in TAA, but contemporary data evaluating survival and cardiovascular outcomes in broad TAA populations are limited. Glucagon-like peptide-1 receptor agonists (GLP-1 RAs) and sodium-glucose cotransporter-2 inhibitors (SGLT2 inhibitors) have established cardiometabolic benefits, yet their role in TAA has not been well defined. Methods: We performed a retrospective multicenter cohort study of adults with imaging-confirmed TAA diagnosed between 1 January 2018 and 1 January 2026 using a Mayo Clinic electronic data platform encompassing more than 15 million patient records. Primary exposures were documented use of RAAS inhibitors, GLP-1 RAs, and SGLT2 inhibitors, evaluated individually and in prespecified combination-therapy analyses. Propensity score matching was used to balance demographics, comorbidities, aortic procedural history, and concomitant cardiovascular medications. Primary outcomes were all-cause mortality and major adverse cardiovascular events (MACE) through 60 months. Results: The study included 162,126 patients with TAA. After matching, RAAS inhibitor use was associated with higher 60-month overall survival (88.3% vs. 85.5%; hazard ratio [HR], 0.79; 95% CI, 0.76–0.83; *p* < 0.001) and MACE-free survival (86.1% vs. 84.2%; HR, 0.87; 95% CI, 0.83–0.91; *p* < 0.001). GLP-1 RA therapy was associated with higher overall survival (97.5% vs. 92.5%; HR, 0.32; 95% CI, 0.27–0.38; *p* < 0.001) and MACE-free survival (93.2% vs. 89.3%; HR, 0.62; 95% CI, 0.56–0.70; *p* < 0.001). SGLT2 inhibitor therapy was similarly associated with higher overall survival (89.8% vs. 81.5%; HR, 0.51; 95% CI, 0.47–0.54; *p* < 0.001) and MACE-free survival (86.3% vs. 79.1%; HR, 0.62; 95% CI, 0.58–0.66; *p* < 0.001). Combination therapy with RAAS inhibitors plus either GLP-1 RAs or SGLT2 inhibitors was associated with incremental improvements in overall survival and MACE-free survival compared with GLP-1 RA or SGLT2 inhibitor monotherapy. Conclusions: In this large propensity-matched TAA cohort, RAAS inhibitors, GLP-1 RAs, and SGLT2 inhibitors were each associated with improved survival and fewer major cardiovascular events, with additional benefit observed for RAAS-based combination therapy. These findings support further prospective investigation of integrated cardiometabolic and vascular-targeted therapy in TAA, while underscoring that observational associations should not be interpreted as proof of aneurysm-specific disease modification.

## 1. Introduction

Thoracic aortic aneurysm (TAA) is often clinically silent until the development of dissection, rupture, compressive symptoms, or incidental detection during imaging performed for another indication [1]. Once identified, management is built around surveillance, control of hemodynamic stress, modification of cardiovascular risk factors, and timely surgical or endovascular intervention when anatomic thresholds or symptoms warrant treatment. Although this approach has improved detection and procedural timing, medical therapy for TAA remains comparatively underdeveloped. Current guidelines emphasize blood pressure control, smoking cessation, imaging follow-up, and selected use of beta-blockers or RAAS inhibitors, particularly in genetically mediated aortopathies, but there remains no broadly accepted pharmacologic strategy proven to improve long-term survival or reduce major cardiovascular events in the general TAA population [2,3].

The biology of aneurysm disease is broader than aortic diameter alone. Aneurysm formation and progression reflect a convergence of wall stress, vascular smooth muscle cell injury, extracellular matrix remodeling, oxidative stress, inflammation, endothelial dysfunction, and systemic cardiometabolic disease. Angiotensin II signaling is central to several of these pathways, including vascular oxidative stress, matrix metalloproteinase activation, inflammatory cell recruitment, and maladaptive transforming growth factor-β signaling [4,5]. These mechanisms provide a biologic rationale for RAAS inhibition that extends beyond simple blood pressure lowering. The clinical evidence for RAAS inhibition in aortic disease has been strongest in Marfan syndrome and related heritable aortopathies. Experimental work demonstrated that losartan could attenuate aneurysm formation in a murine Marfan model, and subsequent clinical trials and meta-analyses have supported a role for angiotensin receptor blockade, often alongside beta-blockade, in reducing aortic root enlargement in selected Marfan populations [6,7,8,9]. Whether similar vascular or prognostic benefits extend to large, real-world TAA cohorts that include predominantly nonsyndromic disease remains uncertain.

At the same time, GLP-1 RAs and SGLT2 inhibitors have reshaped the treatment of diabetes, obesity, chronic kidney disease, and heart failure. GLP-1 RAs improve weight, glycemic control, inflammation, vascular endothelial function, and atherosclerotic outcomes, while major cardiovascular outcome trials have demonstrated reductions in adverse cardiovascular events in high-risk patients with and without diabetes [10,11,12,13,14]. Experimental aneurysm data further suggest that GLP-1 signaling may reduce vascular inflammation, oxidative stress, matrix remodeling, and aneurysm expansion in abdominal aortic aneurysm models [15]. SGLT2 inhibitors also provide cardiovascular and renal protection across diabetic and nondiabetic populations, with favorable effects on blood pressure, arterial stiffness, inflammation, oxidative stress, volume status, and heart failure outcomes [16,17,18,19]. Preclinical and emerging observational data suggest possible protective associations between SGLT2 inhibition and aortic aneurysm growth and progression, although this evidence remains early and largely non-thoracic [20,21].

These overlapping pathways raise a practical clinical question: in patients with TAA, are contemporary RAAS-targeted and cardiometabolic therapies associated with better long-term outcomes? We therefore evaluated the associations of RAAS inhibitors, GLP-1 RAs, and SGLT2 inhibitors, individually and in combination, with all-cause mortality and MACE in a large multicenter cohort of adults with imaging-confirmed TAA.

## 2. Methods

### 2.1. Study Design and Data Source

We conducted a retrospective multicenter cohort study of adult patients diagnosed with TAA between 1 January 2018 and 1 January 2026 across Mayo Clinic sites in Rochester, Minnesota; Jacksonville, Florida; and Phoenix, Arizona, with additional patients contributed by collaborating healthcare institutions using the same electronic data platform. he platform includes more than 15 million patient records and supports the extraction of diagnoses, imaging findings, medications, procedures, outcomes, and relevant clinical covariates from structured electronic health record data, supplemented by artificial intelligence and natural language processing tools to identify and abstract information from unstructured clinical documentation.

The study was approved by the Mayo Clinic Institutional Review Board and was conducted in accordance with the Declaration of Helsinki. Data were de-identified before analysis, and no direct access to identifiable patient information was permitted during the analytic workflow.

### 2.2. Cohort Definition

Eligible patients were 18 years of age or older with imaging-confirmed thoracic aortic aneurysms, including aneurysms involving the ascending thoracic aorta. To improve diagnostic specificity, inclusion required explicit documentation of TAA in the impression section of a computed tomography, computed tomography angiography, or echocardiography report. Patients were excluded if confirmatory imaging was unavailable or if medication data were incomplete. The index date was defined as the date of TAA diagnosis used for cohort entry.

### 2.3. Medication Exposures

The primary exposures were RAAS inhibitors, GLP-1 RAs, and SGLT2 inhibitors. RAAS inhibitors included angiotensin-converting enzyme inhibitors and angiotensin receptor blockers. To minimize immortal time bias, exposure status was anchored to medication use documented at or prior to the index diagnosis date, and no post-index exposure was used to define baseline treatment groups. Patients were not reclassified based on medications initiated during follow-up. Patients were first analyzed according to use versus nonuse of each drug class. Prespecified combination analyses then compared RAAS inhibitor plus GLP-1 RA therapy versus GLP-1 RA therapy alone and RAAS inhibitor plus SGLT2 inhibitor therapy versus SGLT2 inhibitor therapy alone.

### 2.4. Outcomes

The primary outcomes were all-cause mortality and MACE through 60 months of follow-up. MACE was defined as a composite of cardiovascular death, myocardial infarction, and stroke. Follow-up was measured from the index TAA diagnosis date through the occurrence of the outcome, last available follow-up, or 60 months, whichever occurred first.

### 2.5. Statistical Analysis

Propensity score matching was used to reduce measured confounding between medication exposure groups. Separate 1:1 nearest-neighbor propensity score models were constructed for each exposure comparison. Matching was performed using a caliper width of 0.2 times the standard deviation of the logit of the propensity score. Matching variables included age, sex, race, ethnicity, hypertension, chronic kidney disease, hyperlipidemia, diabetes mellitus, prior thoracic or ascending aortic repair or replacement, beta-blocker use, calcium channel blocker use, and relevant non-indexed medication classes. For example, GLP-1 RA and SGLT2 inhibitor use were included as matching covariates in the RAAS inhibitor analysis when they were not the primary exposure being tested.

Each propensity-matched analysis was conducted independently within the full source cohort. As a result, matched cohorts differed across exposure comparisons and should not be directly compared across analyses. Each analysis represents a distinct matched population derived from the same parent cohort using exposure-specific matching criteria.

After matching, baseline characteristics were compared between exposure groups to assess covariate balance. Kaplan–Meier methods were used to estimate overall survival and MACE-free survival through 60 months. Cox proportional hazards models were used to calculate HRs with 95% CIs. Analyses evaluated individual drug class associations and prespecified combination-therapy contrasts. A two-sided *p* value < 0.05 was considered statistically significant. Because treatment assignment was not randomized, all estimates were interpreted as associations rather than causal effects.

## 3. Results

### 3.1. Study Cohort

**Figure 1:**
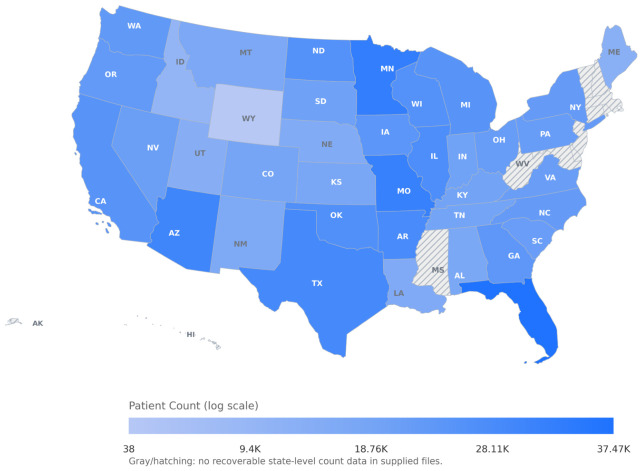
Demographic Distribution of TAA Patients.

The analytic cohort included 162,126 adults with imaging-confirmed TAA. The median age was 63.2 years (interquartile range, 52–72), and the mean age was 60.3 ± 15.3 years. Overall, 75,727 patients (46.7%) were female, and 87.3% were White. Demographic distribution across the United States of TAA patients is presented in Figure 1.

### 3.2. RAAS Inhibitors

Before matching, 72,097 patients were receiving RAAS inhibitors before or at the time of TAA diagnosis, and 90,029 were not. After 1:1 propensity score matching, 33,539 patients were included in each group. Baseline characteristics were well balanced after matching. Mean age was 62.1 ± 13.9 years in the RAAS inhibitor group and 63.2 ± 13.9 years in the non-RAAS group. Female patients represented 44.5% and 44.7% of the matched groups, respectively, and White patients represented 89.8% and 89.6%, respectively.

At 60 months, RAAS inhibitor use was associated with higher overall survival compared with nonuse (88.3% vs. 85.5%; *p* < 0.001), corresponding to a lower hazard of death (HR, 0.79; 95% CI, 0.76–0.83). RAAS inhibitor use was also associated with higher MACE-free survival (86.1% vs. 84.2%; *p* < 0.001; HR, 0.87; 95% CI, 0.83–0.91).

### 3.3. GLP-1 Receptor Agonists

A total of 15,585 patients were receiving GLP-1 RAs before or at the time of TAA diagnosis, compared with 146,541 patients who were not. After propensity score matching, 7834 patients were included in each group. Mean age was 54.4 ± 13.2 years in the GLP-1 RA group and 54.7 ± 14.7 years in the non-GLP-1 RA group. Female patients accounted for 49.7% and 50.3% of the matched groups, respectively, and White patients accounted for 89.4% and 89.3%, respectively.

At 60 months, GLP-1 RA therapy was associated with higher overall survival compared with nonuse (97.5% vs. 92.5%; *p* < 0.001), corresponding to a lower hazard of death (HR, 0.32; 95% CI, 0.27–0.38). GLP-1 RA therapy was also associated with higher MACE-free survival (93.2% vs. 89.3%; *p* < 0.001; HR, 0.62; 95% CI, 0.56–0.70).

### 3.4. SGLT2 Inhibitors

A total of 14,153 patients were receiving SGLT2 inhibitors before or at the time of TAA diagnosis, compared with 147,973 patients who were not. After 1:1 propensity score matching, 13,493 patients were included in each group. Matched baseline characteristics were well balanced. Mean age was 67.4 ± 11.6 years in the SGLT2 inhibitor group and 67.5 ± 11.8 years in the control group. Female patients represented 37.9% and 37.4% of the matched groups, respectively, and White patients represented 90.6% and 90.0%, respectively.

At 60 months, SGLT2 inhibitor use was associated with higher overall survival compared with nonuse (89.8% vs. 81.5%; *p* < 0.001), corresponding to a lower hazard of death (HR, 0.51; 95% CI, 0.47–0.54). SGLT2 inhibitor use was also associated with higher MACE-free survival (86.3% vs. 79.1%; *p* < 0.001; HR, 0.62; 95% CI, 0.58–0.66).

### 3.5. Combination Therapy with RAAS Inhibitors and GLP-1 Receptor Agonists

Before matching, 7891 patients were receiving both RAAS inhibitors and GLP-1 RAs, while 2411 were receiving GLP-1 RAs without RAAS inhibitors. After 1:1 propensity score matching, 1243 patients were included in each group. RAAS inhibitors and GLP-1 RAs, while 2411 were receiving GLP-1 RAs without RAAS inhibitors. After 1:1 propensity score matching, 1243 patients were included in each group. Mean age was 61.2 ± 11.5 years in the combination-therapy group and 61.1 ± 11.2 years in the GLP-1 RA monotherapy group. Female patients represented 58.7% and 58.8% of the matched groups, respectively, and White patients represented 91.8% in both groups.

**Figure 2:**
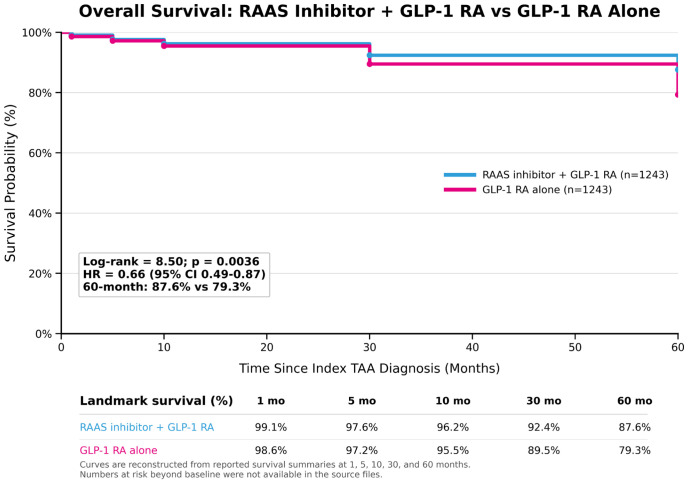
Kaplan–Meier survival curve for patients receiving both a RAAS inhibitor and a GLP-1 RA.

**Figure 3:**
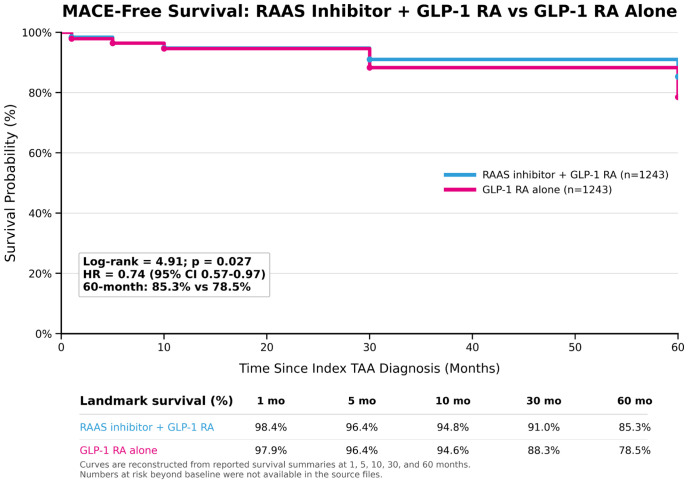
Kaplan–Meier curve for MACE in patients receiving both a RAAS inhibitor and a GLP-1 RA.

At 60 months, RAAS inhibitor plus GLP-1 RA therapy was associated with higher overall survival than GLP-1 RA therapy alone (87.6% vs. 79.3%; *p* = 0.0036; HR, 0.66; 95% CI, 0.49–0.87). Combination therapy was also associated with higher MACE-free survival (85.3% vs. 78.5%; *p* = 0.027; HR, 0.74; 95% CI, 0.57–0.97). These results are presented in Figure 2 and Figure 3, where the blue cohort represents patients receiving both a RAAS inhibitor and a GLP-1 RA.

### 3.6. Combination Therapy with RAAS Inhibitors and SGLT2 Inhibitors

Before matching, 8752 patients were receiving both RAAS inhibitors and SGLT2 inhibitors, while 1366 were receiving SGLT2 inhibitors without RAAS inhibitors. After 1:1 propensity score matching, 741 patients were included in each group. This matched cohort represents a separately derived analytic population specific to the RAAS inhibitor + SGLT2 inhibitor comparison and is not identical to the primary SGLT2 inhibitor vs. nonuser cohort due to independent propensity score matching specifications. Mean age was 67.8 ± 11.5 years in the combination-therapy group and 67.4 ± 11.6 years in the SGLT2 inhibitor monotherapy group. Female patients represented 41.0% and 38.7% of the matched groups, respectively, and White patients represented 92.3% and 92.8%, respectively.

**Figure 4:**
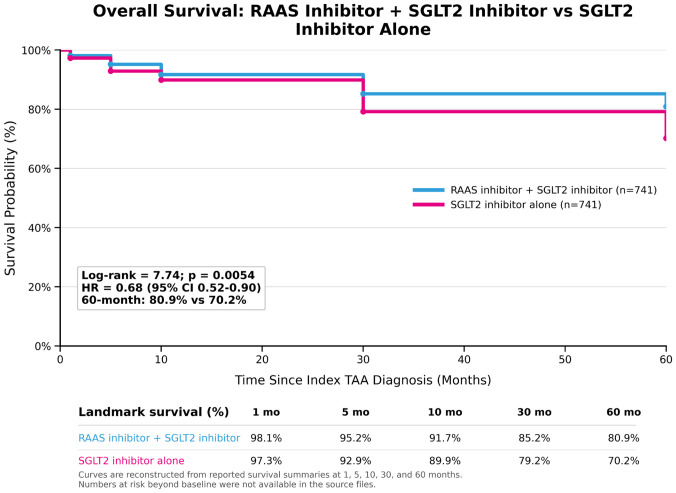
Kaplan–Meier survival curve for patients receiving both a RAAS inhibitor and an SGLT2 inhibitor.

**Figure 5:**
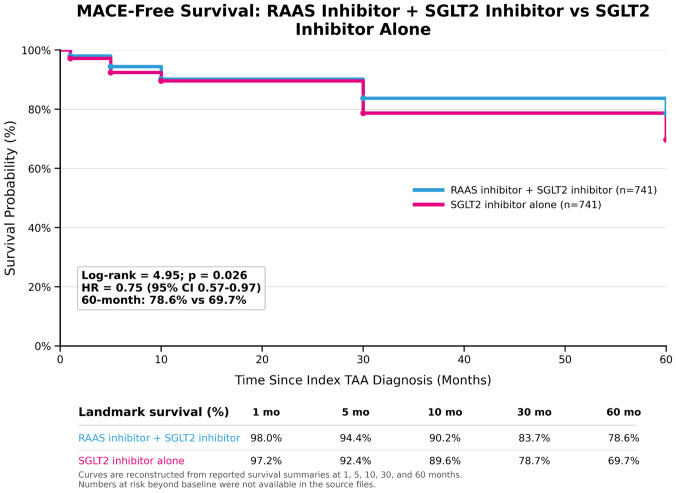
Kaplan–Meier curve for MACE in patients receiving both a RAAS inhibitor and an SGLT2 inhibitor.

At 60 months, RAAS inhibitor plus SGLT2 inhibitor therapy was associated with higher overall survival than SGLT2 inhibitor therapy alone (80.9% vs. 70.2%; *p* < 0.001; HR, 0.68; 95% CI, 0.52–0.90), and these results are presented in Figure 4. Combination therapy was also associated with higher MACE-free survival (78.6% vs. 69.7%; *p* = 0.026; HR, 0.75; 95% CI, 0.57–0.97). These results are presented in Figure 5, where the blue cohort represents patients receiving both a RAAS inhibitor and an SGLT2 inhibitor.

## 4. Discussion

In this large multicenter cohort of more than 162,000 adults with imaging-confirmed TAA, use of RAAS inhibitors, GLP-1 RAs, and SGLT2 inhibitors was consistently associated with higher 60-month survival and higher MACE-free survival after propensity score matching. The findings were directionally consistent across all three therapeutic classes, and RAAS-based combination therapy was associated with additional benefit compared with GLP-1 RA or SGLT2 inhibitor monotherapy. Notably, the magnitude of association observed with GLP-1 RA therapy was greater than that observed for the other therapeutic classes and should be interpreted in the context of the observational study design. The central message is not that these therapies have been proven to slow aneurysm growth, prevent dissection, or replace established surveillance and operative thresholds. Rather, the findings suggest that patients with TAA may derive clinically meaningful prognostic benefit from comprehensive cardiometabolic and vascular risk optimization, and that this strategy deserves a prospective study with aneurysm-specific endpoints.

The RAAS inhibitor findings are biologically plausible and clinically coherent. Angiotensin II has a well-described role in aneurysm biology through oxidative stress, inflammatory signaling, vascular smooth muscle cell injury, extracellular matrix degradation, and dysregulated transforming growth factor-β signaling [4,5]. In Marfan syndrome, losartan and related ARB strategies have been supported by experimental models, randomized clinical trials, and individual-patient meta-analysis, although the magnitude and clinical context of benefit vary across studies [6,7,8,9]. The present analysis extends this rationale to a much broader real-world TAA cohort and suggests that RAAS inhibition may be associated with improved survival and fewer cardiovascular events beyond selected heritable aortopathy populations. Importantly, these data do not prove that RAAS inhibitors directly modified aneurysm biology in this cohort; the observed benefit could also reflect improved blood pressure control, reduced heart failure risk, better renal-cardiovascular management, or residual differences between treated and untreated patients.

The association between GLP-1 RA therapy and improved outcomes was particularly strong, although the magnitude of effect was greater than that observed in most cardiovascular outcome trials and should be interpreted in the context of an observational, nonrandomized treatment design. This finding is clinically interesting because GLP-1 RAs address several conditions that frequently coexist with TAA, including diabetes, obesity, atherosclerotic cardiovascular disease, chronic inflammation, and endothelial dysfunction. Large outcome trials have demonstrated that GLP-1 RAs reduce cardiovascular events in patients with type 2 diabetes, and SELECT extended the cardiovascular benefit of semaglutide to patients with overweight or obesity and established cardiovascular disease but without diabetes [12,13,14]. These observations support the concept that GLP-1 RA benefit is not limited to glucose-lowering. Experimental aneurysm models also suggest that GLP-1 signaling may reduce macrophage activation, oxidative stress, matrix metalloproteinase activity, and aneurysm progression [15]. The current study adds a human TAA signal, but it should be interpreted as hypothesis-generating until studies with medication duration, dose–response, aneurysm size, aortic growth, and dissection or rupture outcomes are available.

SGLT2 inhibitor therapy was also associated with substantially higher survival and MACE-free survival. These agents now have a broad evidence base in heart failure, chronic kidney disease, and diabetes, with mechanisms that include natriuresis, improved ventricular loading conditions, blood pressure reduction, favorable renal hemodynamics, improved arterial stiffness, lower oxidative stress, and anti-inflammatory effects [16,17,18,19]. Several of these pathways are relevant to aortic wall stress and systemic vascular resilience. Preclinical data show that empagliflozin can reduce angiotensin II-induced dissecting abdominal aortic aneurysm formation in ApoE-deficient mice, and recent observational data suggest lower aortic aneurysm risk among patients with type 2 diabetes treated with SGLT2 inhibitors, potentially mediated by inflammatory and oxidative stress pathways [20,21]. These data remain indirect for TAA, but they strengthen the rationale for evaluating SGLT2 inhibitors in structural vascular disease rather than only in conventional cardiometabolic disease categories.

The combination-therapy results are clinically compelling because they mirror a larger shift in cardiovascular medicine: patients with complex vascular disease often benefit from layered, mechanism-diverse therapy rather than from isolated single-pathway treatment. In heart failure, simultaneous targeting of neurohormonal, renal, metabolic, and hemodynamic pathways produces outcome gains that exceed those expected from any one class alone [22]. A similar framework may be relevant to TAA, particularly in older patients and those with hypertension, diabetes, obesity, chronic kidney disease, heart failure, or established atherosclerotic disease. RAAS inhibitors may address vascular remodeling and wall stress, GLP-1 RAs may address metabolic inflammation and weight-related cardiovascular risk, and SGLT2 inhibitors may address cardiorenal and hemodynamic vulnerability. Whether these pathways interact directly within the thoracic aortic wall remains unknown, but the observed combination signals justify dedicated prospective evaluation.

These findings also help reposition how medical therapy in TAA might be studied. Historically, pharmacologic work in aortic disease has focused on aortic diameter, growth rate, and syndromic cohorts, while broader outcomes such as survival and MACE have received less attention. Yet many patients with TAA die from competing cardiovascular causes rather than aneurysm rupture alone. A treatment strategy that improves overall vascular and cardiometabolic health may therefore be clinically valuable even if its direct effect on aneurysm expansion is modest or absent. This distinction matters: the present findings should not be framed as proof of aneurysm stabilization, but they do support a broader disease-management model in which TAA is treated as both an aortic condition and a marker of systemic cardiovascular risk.

### 4.1. Future Directions

Future studies should test these associations in prospectively designed cohorts with detailed medication exposure, standardized imaging, and aneurysm-specific outcomes. Key endpoints should include aortic growth rate, dissection, rupture, aortic repair, aortic-related mortality, and competing cardiovascular events. Studies should also evaluate whether treatment effects differ by aneurysm location, baseline diameter, genetic or syndromic status, bicuspid valve status, diabetes, obesity, chronic kidney disease, and background beta-blocker or RAAS inhibitor therapy. Mechanistic studies using circulating inflammatory markers, vascular imaging, and aortic tissue analyses may help determine whether the observed clinical associations reflect direct aortic wall effects, systemic cardiovascular risk reduction, or both.

### 4.2. Limitations

Several limitations should be emphasized. Firstly, this was an observational retrospective study, and causality cannot be established despite propensity score matching. Residual confounding, confounding by indication, healthy-user effects, access-to-care differences, medication adherence, and differences in follow-up intensity may have influenced the observed associations. Secondly, medication exposure was based on documented active use before or at the time of TAA diagnosis; dose, duration, persistence, discontinuation, and postdiagnosis treatment changes were not fully captured. Thirdly, although the cohort required imaging confirmation, granular aneurysm characteristics such as maximal diameter, growth rate, aortic segment, morphology, genetic status, family history, and detailed operative thresholds were not uniformly available. Fourthly, the primary outcomes were all-cause mortality and MACE rather than aneurysm-specific endpoints. As a result, the study cannot determine whether these therapies slowed aortic enlargement, reduced dissection or rupture risk, delayed operative intervention, or reduced aortic-specific mortality. Additional limitations relate to outcome ascertainment and cohort heterogeneity. MACE was defined using available electronic data, and cardiovascular death classification may be imperfect in retrospective datasets. The cohort likely included a mixture of syndromic and nonsyndromic TAA, degenerative aneurysm disease, bicuspid aortopathy, postsurgical patients, and patients with variable imaging indications. Although procedural history and measured comorbidities were included in matching, unmeasured clinical severity may remain. Finally, the large sample size increases statistical power but does not eliminate bias; very small absolute differences can achieve statistical significance, and effect estimates should be interpreted alongside absolute event rates, biologic plausibility, and the limitations of nonrandomized treatment assignment. Additionally, the markedly favorable associations observed for GLP-1 RAs and SGLT2 inhibitors may reflect residual treatment-selection bias, differential healthcare engagement, or survivor bias despite propensity score matching procedures.

### 4.3. Conclusions

In a large propensity-matched cohort of adults with imaging-confirmed TAA, RAAS inhibitors, GLP-1 RAs, and SGLT2 inhibitors were each associated with higher survival and higher MACE-free survival through 60 months. RAAS-based combination therapy with either GLP-1 RAs or SGLT2 inhibitors was associated with additional benefit compared with cardiometabolic monotherapy alone. These findings support a more integrated view of TAA care in which aortic surveillance and operative decision-making are complemented by aggressive cardiometabolic and vascular risk optimization. Prospective studies with aneurysm-specific outcomes are needed before these associations can be translated into disease-modifying treatment recommendations for TAA.

## Data Availability

The data presented in this study are available on request from the corresponding author, consistent with institutional policies and applicable privacy regulations.
